# Experiences of people living with HIV in low- and middle-income countries and their perspectives in self-management: a meta-synthesis

**DOI:** 10.1186/s12981-024-00595-7

**Published:** 2024-01-31

**Authors:** Tegene Legese Dadi, Anja M. C. Wiemers, Yadessa Tegene, Girmay Medhin, Mark Spigt

**Affiliations:** 1 School of Public Health, College of Medicine & Health Science, Hawassa University, Hawassa, Ethiopia; 2 Faculty of Health, Medicine and Life Sciences, Care and Public Health Research Institute (CAPHRI), Maastricht University, Maastricht, Netherlands; 3 Aklilu Lemma Institute of Pathobiology, Addis Ababa University, Addis Ababa, Ethiopia; 4 MERQ Consultancy PLC, Addis Ababa, Ethiopia; 5 General Practice Research Unit, Department of Community Medicine, UiT the Arctic University of Norway, Tromsö, Norway

**Keywords:** HIV, PLWH, Self-management, Meta-synthesis, Low- and middle-income country

## Abstract

**Introduction:**

Availability of anti-retroviral treatment has changed HIV in to a manageable chronic disease, making effective self-management essential. However, only a few studies in low- and middle-income countries (LMICs) reported experiences of people living with HIV (PLWH) on self-management.

**Methods:**

This meta-synthesis of qualitative studies investigated perspectives of PLWH in LMICs on self-management. Various databases, including PubMed, EMBASE, EBSCO, and CINHAL, were searched through June 2022. Relevant additional articles were also included using cross-referencing of the identified papers. We used a thematic synthesis guided by the "Model of the Individual and Family Self-Management Theory" (IFSMT).

**Result:**

PLWH in LIMICs experience a variety of challenges that restrict their options for effective self-management and compromises their quality of life. The main ones include: misconceptions about the disease, poor self-efficacy and self-management skills, negative social perceptions, and a non-patient-centered model of care that reduces the role of patients. The experiences that influenced the ability to practice self-management are summarized in context (the condition itself, physical and environmental factors, individual and family factors) and process factors (knowledge and beliefs, relationship with the health care worker, self-regulation skills and abilities, and social facilitation). Context and process greatly impacted quality of life through the self-management practices of the patients.

**Conclusion and recommendation:**

PLWH encounter multiple challenges, are not empowered enough to manage their own chronic condition, and their needs beyond medical care are not addressed by service providers. Self-management practice of these patients is poor, and service providers do not follow service delivery approaches that empower patients to be at the center of their own care and to achieve an effective and sustainable outcome from treatment. These findings call for a comprehensive well thought self-management interventions.

**Supplementary Information:**

The online version contains supplementary material available at 10.1186/s12981-024-00595-7.

## Introduction

HIV (human immunodeficiency virus) affected close to 38 million adults and children worldwide by the end of 2020 [1]. Fortunately, the emergence of antiretroviral treatment (ART) and its availability has turned HIV from a life threatening disease into a manageable chronic disease [2]. This has resulted an emergence of new challenges that include long-term medication use and associated complacency, and multi-morbidities. This has necessitated a shift from provider centered health care services to self-management (i.e., how patients manage their illness) practices as part of patient care practices. This practice is crucial for chronic diseases management, as only patients can oversee the day-to-day care of themselves. Self-management practice among PLWH consists of medical management; maintaining, changing or creating healthy behaviors; and dealing with the emotions of living with the chronic disease [3]. The practice of self-management requires a set of skills such as problem solving, decision making, resource utilization, forming a patient/health care provider partnership, and action taking [4].

The effects of programs to improve self-management have been studied in randomized controlled trials (RCTs) in high income countries [5–7]. In one of these studies showed that self-management programs for PLWH had significant short-term improvements in physical, psychosocial, health literacy, and behavioral outcomes (8]. Mobile-based self-management interventions, though feasible and effective, are usually focused on a very limited aspect of self-management, e.g., reminders to improve medication adherence [9]. The most promising outcomes were achieved in HIV self-management programs that comprised a combination of skill training, phone counseling, counseling with symptom management manuals, and technology assisted interventions [8).

Though the need to practice self-management in the service delivery is crucial for patients and the health system, its practice in the health care system and research on its modality of implementation in low- and middle- income countries (LMICs) is limited [5–8]. In these countries self-management interventions could have a great impact, because it is less expensive, but potentially more effective than traditional provider-centered chronic care [10]. In order to come up with acceptable and effective interventions for PLWH in LMICs, an understanding of the components of self-management and the perspectives of patients towards self-management intervention is important. The evidence from high income country setting showed that the "Individual and Family Self-Management Theory" (IFSMT) offers a good model of factors that influence self-management [11].

The purpose of this paper is to report synthesized understanding of the perceptions and experiences of HIV patients involved qualitative studies [12]. Taking the patients' point of view has been shown to improve the efficacy of self-management interventions [13, 14]. While there are multiple studies on self-management as perceived by PLWH in high income countries [5–7], there is no synthesized evidence in developing countries that can be utilized by the health care providers. Therefore, in order to develop a starting model for future interventions, the study's aim is to determine the perceived factors that PLWH in LMIC believe affect self-management and their experiences.

## Methodology

### Study design

We followed the steps of meta-synthesis described by Sandelowski and Barroso [15] to summarize the findings from published qualitative studies. This approach is suitable to synthesize qualitative literature without simplifying it, taking into account the diversity of theoretical and methodological approaches.

### Search strategy and databases

A comprehensive search strategy was used to identify existing literature on experiences of PLWH on self-management. The search was conducted in June 2022 using Embase, Pubmed, Google Scholar, CINHAL, MEDLINE, Psych info and the Cochrane database. The search terms included “people living with HIV/ HIV patients/PLWH/ HIV” and “self-management/selfmanagement/self-care/self-evaluation/ self-monitoring” and “interviews/interview/questionnaire/observational study/focus group/ focus groups” (Additional file 1). Moreover, hand search was conducted through cross referencing of the identified papers. The list of final citations was saved in the endnote and screened for duplicates and further processing.

### Screening of identified articles

All potential articles underwent a two-stage screening based on inclusion and exclusion criteria (Table 1). After removing duplicates, articles were screened based on title and abstract to determine eligibility for inclusion. After the initial screening, the full texts of each article were read in full and assessed for inclusion. The references of the included studies were further reviewed to identify for any missed eligible papers and to include the eligible ones. The screening process was carried out by two authors of this paper (AW & TLD). A random sample of 50 articles from the title and abstract screening and all articles from the full text screening were reviewed by a third author (YT) for quality assurance to check the relevance of the papers. Disagreements on the relevance of the papers were discussed until a consensus was reached.

**Table 1:** Inclusion and exclusion criteria of articles

Inclusion criteria	Exclusion criteria
Qualitative studies on the perceptions of PLWH, study participants above the age of 18, addressed on self-management	Unrelated studies, such as: - studies on HIV prevention - studies that focus only on adolescence living with HIV - studies whose conclusions on self-management is based on regression analysis
Studies conducted in low-middle income countries as per World Bank classification	Abstract papers such as preceding papers, conference, editorial, and author response letters and books
No restrictions regarding race, gender and date of publication	Quantitative studies, case reports, case series and reviews of quantitative studies
	Studies written in languages other than English

### Critical appraisal of identified papers

We carried out a quality appraisal using the Critical Appraisal Skills Programme (CASP) checklist [16] to assess the methodological strengths and limitations of the included studies. One author (AW) carried out the appraisal, and the second author (TLD) checked the assessments. In case of disagreement between the two authors, the issues were resolved through discussions.

### Approach of data extraction and meta-synthesis

The data extraction was conducted using a template prepared in Microsoft Excel. This data extraction templates includes relevant study information that include study design, key findings, and conclusions, as well as indicators of the self-management perspectives of PLHIV in the form of quotes. Data extraction was carried out independently by AW and TLD, with disagreements being discussed and settled. Line-by-line coding was done on the texts using an inductive approach, followed by grouping of codes that had similar concepts into themes. The final stage involved developing analytical ideas that went "beyond" the content of the original. Through debate, more topics were developed from the individual analytical themes.

**Fig. 1:**
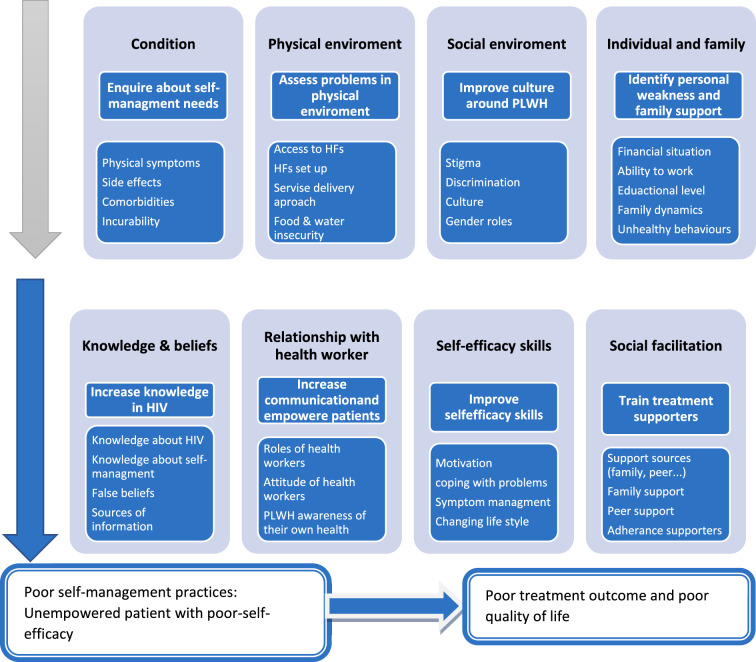
Adjusted “Model of the Individual and Family Self-Management Theory” for PLWH [11]

The coding of texts and thematization were conducted using Nvivo version 12 qualitative software. The themes were then compared to the dimensions and sub-dimension of the “Model of the Individual and Family Self-Management Theory” (IFSMT) [11]. This model is suitable to approach self-management among HIV patients through individual which is focusing on patients only and family lens which focus on patient while considering the family, friendship network, and community relationships that potentially affect the health status of the patient. The model is comprehensive and can address multidimensional areas of the patient during service delivery. The model has three dimensions (see Fig. 1): context, process and outcomes. The context consists of risk and protective factors that relate to condition, social and physical environment and individual and family factors. The process of self-management is said to be determined by knowledge and beliefs, self-efficacy and social facilitation. The outcomes of treatment can be divided into proximal outcomes such as adherence to medication or distal outcomes such as quality of life.

Differences between the IFSMT model and the themes that emerged from the literature were observed. Changes were made accordingly and the IFSMT model was adapted to create a clear overview of the self-management perspectives of PLWH in LMICs and possible self-management intervention goals. After reciting the differences between themes developed from text coding and the dimension of the model, the thematic analysis was conducted using the dimensions and sub-dimensions of the IFSMT model. Even though the model has context, process and outcome dimensions, we only report on the first two dimensions. Since most of the identified articles did not include outcomes, we chose not to include detailed descriptions of proximal and distal outcomes into our model. We have four themes in each of the context and process dimensions (Fig. 1).

## Results

**Fig. 2:**
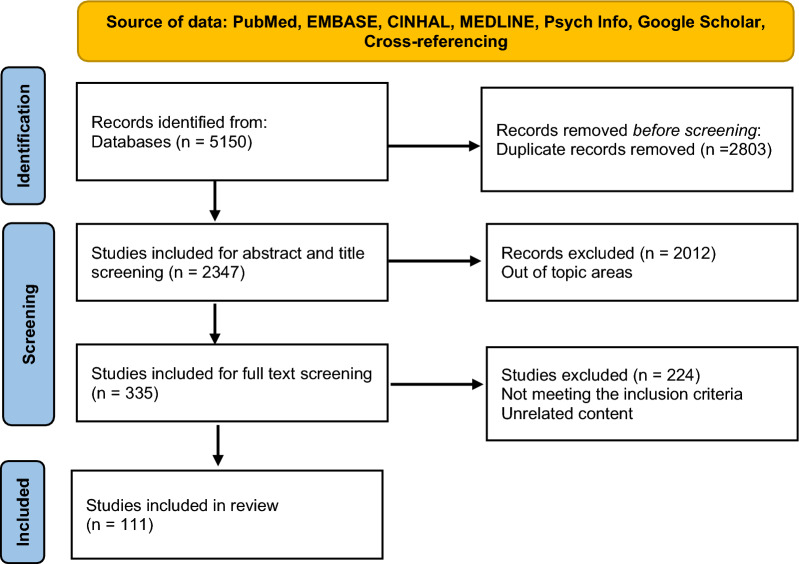
PRISMA flow chart of study selection

The search strategy identified 5150 papers, 2803 articles were excluded as duplicates and 2012 articles were excluded during title and abstract review. Of the remaining 335 papers, a total of 111 papers on PLWH perceptions of self-management in LMIC were identified and included in the review (Fig. 2). Of the included studies, 70% were conducted in Africa, 26% in Asia, 3% in South America and 1% in North America. The quality of the included articles was moderate.

### Context dimension

The context related experiences of PLWH were organized into four themes: condition specific, physical environment, social environment, and individual and family themes.

#### Condition specific

PLWH suffered from a wide range of physical symptoms, including fatigue, weight loss, pain, stiffness, itching, dyspnea, and gastrointestinal and gynecological problems [17–30]. These symptoms made it difficult for PLWH to work and perform their daily activities. Comorbidities such as tuberculosis and noncommunicable diseases added to the burden. PLWH often feel better after starting ART; however, many patients experience significant side effects from the medication. Long-term side effects include osteoporosis, changes in cholesterol levels, liver disease, lactic acidosis, and others. Minor side effects include headache and fatigue [17, 18, 20, 21, 26, 30–52]. Medication side effects and the burden of taking multiple pills per day have been cited as barriers to ART adherence [17, 19, 30, 40, 49, 50, 53–57].

*“I sometimes stop taking my medications, because of their side-effects; they make me aggressive. I treat my child so badly for saying anything at all. That's why I stop taking them, it's for my kid's sake. My double-dose pills give me diarrhea.” *[58]

*“The pills ... they make me feel like sleeping ... at work I feel like sleeping ... at times I skip pills for two days and take them during weekend.” *[43]

PLWH initially resist to accepting their diagnosis of HIV [46, 59]. This is partly or fully linked to the incurability of the disease and its stigma, loneliness, sadness and suicidality that often lead to psychological problems. These disease-specific issues make self-management practices more challenging. The following quote illustrates the perspective of the disease:

*"I feel like why should I live in this world, or what am I living for? Sometimes I get completely down, emotionally depressed. Sometimes I even feel like killing myself or committing suicide *[60]

#### Physical environment

The physical environment has a major impact on patients' ability to self-manage, which is more pronounced in LMICs. The greatest barrier to self-management was limited access to healthcare facilities due to long distances to the clinic. Because specialized HIV clinics were not available within reasonable distances, patients often had to travel long distances for their appointments [20, 21, 34, 39, 42, 50, 56, 61–71]. Other problems with health facilities include a lack of integration of services, which is reported mainly by PLWH with co-morbidities (19, 26, 72]. Another problem was that the design of the facility didn't provide privacy for PLWH [65, 68, 70, 73].

"I came from very far, over 50 km from here. Before coming to the hospital, I had to plan the money for the trip to the clinic. In fact, my extra medicine was finished yesterday. [11]

"The problem is the location of the clinic. The waiting area is in an open place, so whoever goes there will know that they are infected with HIV, but some of us have not disclosed our status for fear of losing respect...., that really discouraged me from coming” [70]

The organization of HIV care affects self-management: PLWH complained about long waiting times, short consultations with providers, and the risk of postponing appointments, often due to a shortage of health care workers [39, 52, 55, 71]. Lack of certain necessary diagnostic materials (e.g., CD4 test not available) and drugs (e.g., ART out of stock) are often mentioned [19, 21, 51, 52, 61, 65, 74, 75].

PLWH may also face other non-medical barriers to self-management: unstable housing and water insecurity [22, 50, 62, 76) and food insecurity [21, 33–37, 40, 50, 69, 77–85]. Taking ART without food exacerbates side effects and leads to lower adherence. PLWH called for support in the form of skills training and education to help them cope with their problems and also to relieve pressure on the health system. Some illustrative quotes:

*"If you want to enjoy taking these pills and have them help you, you need to have some food. If you have some food, then there is no other problem."  [*77]

*"They tell us to eat fruits and vegetables and here in Dares Salaam you have to buy all these things. Maybe they should give us some help. Right now, you can go a day without eating and you cannot even afford an orange. *[33]

#### Social environment

Our literature review shows that the social environment has a huge impact on the self-management of PLWH. HIV is a highly stigmatized disease, which makes PLWH very reluctant to disclose their status. Fear of disclosure inhibits self-management in many ways [21, 22, 29, 31, 36, 37, 39, 40, 42, 45, 46, 49, 50, 58, 62, 73, 86–90]. PLWH typically wait a long time before being tested, do not take their medications on time, isolate themselves from their social environment, do not attend the clinic regularly, or travel to more distant clinics to avoid disclosure.

PLWH reported being treated differently after their co-workers and employers found out about their HIV status, resulting in outright discrimination in their communities and workplaces. In some cases, disclosure led to dismissal from work. Individuals reported missing appointments because of fear of disclosure or avoiding regular sick leave [43, 57, 73, 89). Discrimination against PLWH by health care workers was also reported [22, 26, 60, 61, 65, 88, 91]. This is harmful because it makes people afraid to seek help.

*"When people out there find out that you are HIV positive, they treat you badly. Sometimes, if you are employed, you may lose your job if you disclose your status. Even in the family, if you disclose that you are HIV positive, your people may stop eating with you, they just ignore you, saying that you are useless because you can die any time. You can even be denied a loan from the system because of your sero status—they claim that you can die any time and therefore default" *[18]

*"Some of the doctors and even counselors wear masks when they talk to an HIV-positive person. They also turn their faces away from us. If doctors treat us with such indifference, how can we expect others to be considerate?" *[92]

While disclosure can lead to social support, it can also lead to harassment. PLWH struggled with alienation from partners, experienced verbal harassment and violence, and were excluded from activities [50, 63].

*"When I told my husband, he didn't believe me. He just started laughing and told me I was going to die this year." *[71]

*"She [the sister] did not visit me again after I disclosed. I regretted it, I really regretted it. From now on, I will not disclose to anyone." *[30]

Culture in general, and gender inequality in particular, seemed to have a major impact on the care and support of people with HIV. Gender inequality seemed to reduce self-management [31, 36, 42, 82]. In most developing countries, health issues are seen as women's issues, which makes men reluctant to engage in self-care and visit a clinic. On the other hand, this has also led to a lack of support for women, as men who help their wives (for example, by accompanying them to their PMTCT appointments) are perceived as weak [31, 36, 82]. The cultural belief that caregiving is weakness was particularly damaging because women with HIV were often financially dependent on their husbands. Husbands who did not see the value of self-management could even prevent women from practicing self-management [31, 36, 40, 42, 70, 71, 93, 94]. Examples include refusing to use a condom, not providing money to travel to a health facility, or forbidding their partner from using ART. Community-based problems interfere with proper self-management, leading to poorer treatment outcomes.

*"If a woman is HIV positive, people think she was a prostitute. ...and then these [women] are discriminated against. ...if they are known to be on ART. So, women are ashamed to participate in PMTCT or HIV testing for fear of being shamed." *[36]

#### Individual and family

How well HIV is managed depends on a person's or family's characteristics. The patient's work status, financial situation, level of education, family dynamics, and unhealthy behaviors are some examples of individual and family behaviors that affect the patient's state of self-management and treatment outcomes [21, 29, 39, 78, 80, 89, 95, 96]. The ability to work is very important; it was seen as a source of pride and purpose for PLWH and was sometimes used as a coping mechanism [21, 89, 96]. Not being able to work meant having to rely on others for financial support. The financial situation, combined with the cost of care, has a significant impact on self-management [17, 23, 26, 30, 32, 34, 38, 39, 42, 45, 54, 55, 65, 72, 79, 80, 89, 91, 97–99]. For example, if a family or person cannot afford to travel to the clinic or to buy medication, this will negatively affect their adherence to ART treatment.

*“Working for myself has helped me so much during this period of “sickness” and I don’t worry about anything.”  *[80]

Patients' level of education has a significant impact on their ability to process information and solve problems, which affects their adherence to treatment [31–33, 38, 45, 50, 53, 67, 71, 72, 77, 100]. Illiterate PLWH struggled with reading and writing but did not always seek help [45, 84]. Families with less education struggled to accept the HIV status of a family member, and as a result of their understanding, they were more fearful and prone to misconceptions.

*"I don't read books about HIV because I can't read them in my office or at home because I live with other people. If they saw me reading those books, they would know my situation. So now I only get information from my providers when I go to pick up my medication.” *[30]

Family dynamics and changes in life style influence both self-management and treatment outcomes. When PLWH have to care for the family, they often do not prioritize themselves and do not make time for symptom management or emotional coping. Lifestyle changes can also be challenging because they sometimes mean that the whole family has to adapt. For example, food was often shared with the entire family, which meant that the entire family had to adapt to a new diet that was appropriate for the household member with HIV [19, 42, 63, 76, 83, 93]. Unhealthy lifestyles of individuals or their families, such as addiction or stress, alcohol use, lower adherence and their ability to self-manage [43, 46, 53, 58, 67, 77, 82, 90]. The following are some illustrative quotes:

*"I went to the village in December and when it was time to come back before my appointment on January 3, 2011, I could not come back. After a few days I lost another relative and so I stayed in the village. I had planned to return on January 1 for an appointment at the clinic, but I did not return. " *[63]

*"I have also stopped drinking so that I can concentrate on my treatment [...] I take this program seriously because my family benefited from it." *[82]

### Process dimension

Knowledge and beliefs, relationships with healthcare professionals, self-efficacy skills, and social facilitation skills are the four categories that are organized under the process dimension.

#### Knowledge and beliefs

We find that PLWH see a clear link between knowledge and self-management [17, 19, 28, 30, 31, 34–37, 39, 40, 42, 44–46, 49–51, 55, 57, 65, 67, 71, 72, 76, 80–82, 84, 85, 88, 94, 95, 100–104]. Sometimes people are unaware of their diagnosis, what exactly HIV is, what ART is, and how to take ART [23, 36, 39, 43, 65]. People first need to understand their disease and the need for treatment, then they will be able to put the advice given into context and will be more likely to adhere to it. Knowing that HIV can be controlled with medication helps with acceptance and empowerment. PLWH themselves say that when they are informed about side effects, they are more likely to adhere to the medication because they know that the side effects they experience are normal.

*“I don’t know what those tablets help; you are just given and you swallow them” *[65].

*“We need help to be healthy ... We need more health information and support for being healthy.” *[62]

In addition to understanding their disease and its treatment, it is important for them to learn about self-management. PLWH need to be made aware of the need for self-management and the concept behind it so that they can act on it. It is important to teach them about healthy eating, symptom management and where to get help if needed. For example, there are clinics with peer support groups, but PLWH are often unaware of them or do not know how to access them. Some patients rely solely on information provided by health care workers [30, 39]. Others get information from the pharmacist, the community, peers, the radio, and the Internet. Information from the community and the Internet is often misleading, scaring PLWH or leading to misconceptions.

There are many misconceptions and false beliefs about HIV. A common belief is that God can heal HIV and will give a sign when ART is needed. Another common misconception is that ART is ineffective or even harmful. Some other PLWH felt that ART had cured them and stopped taking their medication [32, 33, 35, 38–40, 43, 44, 46, 47, 52, 59, 84, 90, 98, 100, 105]. Another misconception about treatment is that PLWH use herbs instead of ART drugs, believing that herbal use is as effective as ART with fewer side effects. There are PLWH who use herbs in addition to ART for symptom management; in most cases, they are opposed to ART [59, 106, 107]. These misconceptions significantly reduce adherence to ART, and even prevention of mother-to-child transmission has been negatively affected. Illustrative quotes:

*"I stopped taking my medication while I was being baptized with holy water (in a monastery). The priests there told us to just get baptized with the holy water and stop taking antiretroviral pills; I believed in Jesus and just took the holy water to be healed". *[37]

*"I have been using 'imbiza' (traditional medicine) since I stopped taking ARVs..., it was my mother who said we must try the traditional healer because they said his 'imbiza' helps..." *[52]

#### Relationship with health care worker

Relationships between PLWH and health care providers play a critical role in improving service delivery, changing attitudes of PLWH, helping PLWH to take charge of their own care, and influencing PLWH's response to treatment and ability to self-manage their condition [18, 21–23, 26, 28, 31, 36, 39, 42, 44–46, 50, 51, 55, 56, 61, 65, 70, 75–77, 80–86, 88, 91, 94, 95, 98, 100, 103, 106, 108–111]. According to PLWH, health care professionals play a variety of roles, but the most important include providing information, managing symptoms, counseling patients on social and emotional issues, and inspiring and supporting self-management by showing patients how to adopt healthy lifestyles and cope with specific challenges [45, 56, 61, 65, 84, 85, 91, 94, 98]. PLWH valued health workers' knowledge and followed their advice. Trust in the health care worker made it possible to share concerns and receive needs-based treatment [42, 44, 65, 77, 100, 103, 108]. However, the results showed that the relationship with health workers was not always good. A bad experience with a health worker made it difficult for patients to return to the clinic and communicate openly [52, 63, 65, 72, 101].

Many patients reported an initial mistrust of the health care worker, but this was overcome when they saw that the health care worker cared about them. PLWH want a professional who treats them with respect and empathy [19, 28, 35, 37, 38, 47, 60, 61, 63, 65, 68, 69, 72, 73, 88, 100, 102, 110]. It was important for the health worker to be forgiving if the PLWH missed an appointment or could not follow through with advice. Otherwise, the person will be afraid to return to care and discouraged from trying again. In order to discuss the important issues at hand, the health care worker is expected to have sufficient time for the patient. Confidentiality is essential for the patient to speak openly [36, 39, 65, 68, 75, 76, 86, 91].

In low- and middle-income countries, patients often view health care providers as authorities [35, 81]. Many PLWH are not aware that they can ask the healthcare provider about their condition and treatment. If they do not understand what the provider is saying, or if they need help with a particular issue, such as side effects, they do not want to bother the provider [39, 50, 65, 70, 102]. Being able to ask questions and communicate about problems is an important patient responsibility, but many do not seem to be aware of it. The lack of communication leads to a lack of appropriate care and misunderstandings. PLWH appreciate learning about their role and enjoy being taught how to use communication tools such as a body map, pain scale and side effect checklist [102]. The following are some of illustrative quotes:

*"Before, I didn't know if I could ask the doctor about my blood condition because I didn't know if I could ask doctors questions. Now I know that when I come to the hospital, I can ask any question I want" *[102]

#### Self-efficacy skills and abilities

**Fig. 3:**
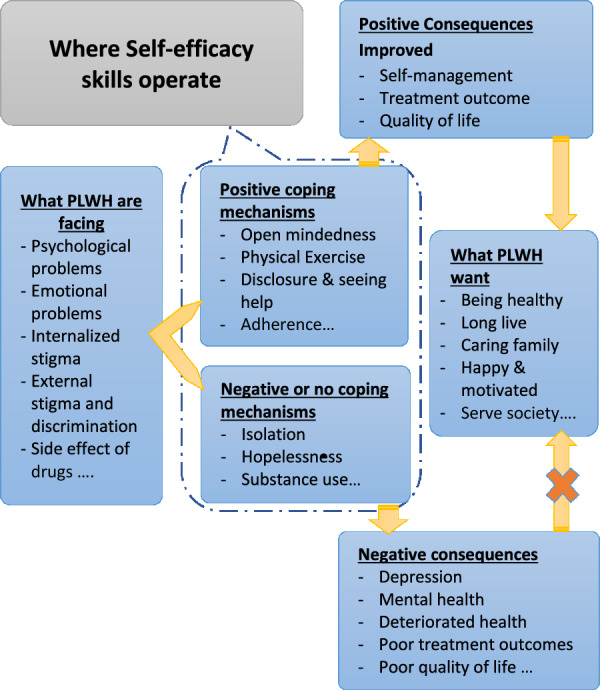
Summary of perspectives of PLWH on “self-efficacy skills and abilities″ their outcomes

Self-efficacy is the process of achieving positive outcomes and fulfilling a desire or plan by controlling one's responses and reactions, including thoughts, emotions, and actions. As presented in the previous sections, PLWH face a multifaceted problem, either from the external environment (family, community, health facility) or internal source, which will necessitate to have the skills of self-efficacy for better self-management practice and treatment outcomes. As summarized in Fig. 3, although PLWH face different problems, they all want to live longer, take care of their families, contribute to society and be healthy through improved self-management practices, treatment outcomes and quality of life [21, 22, 30, 31, 33, 35–39, 42–45, 48, 50, 51, 54, 55, 57, 58, 61, 65, 68, 72, 75, 77, 78, 80, 82, 86, 89, 90, 95–97, 100, 103, 110, 112]. Having such desires or plans is taken as motivation to derive responses and coping mechanisms of PLWH, but some others fail and take a negative path which then ruins their lives. To cope with the internal and external problems, PLWH may have different self-efficacy for problems, positive or negative coping mechanisms (Fig. 3).

Healthy coping mechanisms such as spirituality, physical exercise and changing mindsets are used by many PLWH. Shifting their perspective to a positive one, rationalizing the situation and living in the moment were seen as processes that help with acceptance of their condition. PLWH reported that disclosure made coping easier, as their internal processes could then be shared with the outside world [21, 23, 26, 45, 96, 106, 109, 113].

In order to live a long and healthy life, patients not only have to learn to deal with the emotional and physical problems they experience, but they had to change their lifestyle to prevent decay from the disease or getting co-morbidities. A healthy diet was perceived as important as well as safe sex practices [21, 23, 39, 45, 50, 56, 59, 67, 69, 81, 83, 94, 109]. Starting a new lifestyle can be stressful, and PLWH prefer to have some support in the process. An important step in the process is to negotiate the lifestyle rules to fit one's lifestyle with one's family. PLWH must learn to prioritize their health, but they must do so on their own terms [17, 23, 31, 33, 35, 36, 47, 52, 66, 72, 80–82, 86, 100, 101]. PLWH should be made aware of their role in managing their disease and improve their self-efficacy.

Another important self-efficacy skill is symptom management and adherence. The most important preventive measure is adherence to ART through self-compassionate activities such as yoga, relaxation, daily thoughts or daily exercise were found to be helpful [19, 42, 45, 50, 56, 57, 69, 100]. PLWH use a variety of strategies to increase adherence: reminders, daily routines, having a caregiver, carrying medication with them at all times [17, 19, 30, 35–38, 42, 45, 46, 49, 50, 55–57, 61, 69, 77, 81, 83, 85, 100, 109]. Ways to avoid side effects were also mentioned, for example, side effects were less likely to occur when taken with water and a meal, so PLWH made sure to have these available.

*"It is a matter of convincing myself. I have the confidence and the power to treat myself. My self-efficacy increases from time to time" *(45)

Not all coping strategies are positive. Some people resort to unhealthy habits such as alcohol and drug abuse [42, 56, 89, 90]. Others neglect the diagnosis or blame others for the infection [43, 59, 82, 94].

*"One man chose not to take his medication on Friday evenings because he wanted to go out with friends and drink beer: this selective 'non-adherence' was part of his own self-management and search for well-being, balancing health and social life" *[80]

#### Social facilitation

PLWH need multiple types of support including social, emotional, and adherence support from family, peers, health care providers and the community. These supports help a person adapt to new condition, combat stigma and discrimination, adhere to treatment protocols, and provide financial support and other benefits [19, 25, 31, 53, 61, 96, 97, 99, 104, 111, 113, 114]. Emotional support, for example, is very important in helping people accept their health status and adjust to the "new normal". People appreciate being able to share their feelings and are grateful to be accompanied to clinic visits [25, 31, 45, 56, 61, 77, 80, 97, 99, 114].

The social environment facilitated self-management through financial support [17, 21, 30, 36, 38, 39, 53, 59, 70, 75, 77, 85, 96, 99, 103, 104, 113, 114]. Sometimes social support helped PLWH with their daily tasks. In this way, PLWH are able to access the care they need and have time to practice self-management [25]. Social support often came from partners, family and friends. Community support encouraged PLWH to disclose their HIV status [115). Most often, being open about something resulted in social support rather than prejudice.

*"I have attended both a social support group and a spiritual support group. These groups are very helpful, not only in building a social network of people with similar problems, but also as a safe place to get support, I think" *[25]

''My girlfriend is the only person I have disclosed to, and she has helped me to cope with the disease and to take my HIV medication on the right schedule.'' [115]

Treatment supporters were said to improve the overall health of PLWH [53, 85, 100, 101, 104, 113, 114]. Their main purpose was to ensure that PLWH take their ART, but the role often extended to emotional support, help with daily tasks and dealing with stigma. It should also be noted that although most treatment supporters were reliable, not everyone considered their role to be equally important [53].

"A treatment supporter should be there all the time. Today you are happy, tomorrow you are not; and if someone is not there for you or doesn't really understand you, they will never help you.” [53]*.*

Another very important social facilitator is peer support [25, 39, 42, 45, 49–51, 54, 56, 57, 59–62, 66, 69, 71, 72, 75, 80–82, 87, 88, 90, 94–96, 103, 108, 113, 114]. PLWH described how they saw their peers as role models for adherence and how seeing them looking healthy encouraged them. Many older PLWH also describe pride in being a role model for other people with the disease. Peer support groups provided a sense of belonging, a place to check on each other outside of meetings, and a source of information. PLWH would give each other advice on disclosure, dealing with stigma, healthy eating and ways to improve adherence.

## Discussion

PLWH are caught between their desire to live a healthy, productive, and quality life and the multiple problems arise from both their internal (poor understanding of their disease and its treatment, lack of disclosure, internalized stigma) and external (stigma and discrimination, lack of family or community support, lack of patient-centered care) environments. PLWH respond to a variety of internal and external problems with positive or negative coping mechanisms, which also determine their level of self-management, treatment success and quality of life. Healthcare providers tend to practice professional-centered care and lack an understanding of patients' issues beyond their medical problems. Patients want to overcome the multidimensional problems they face and then live healthy lives by acquiring relevant knowledge and skills to be at the center of their own care management (self-management).

The current meta-synthesis showed that PLWH are situated between their desire to live a healthy, productive, and quality life and the many problems that arise from both their internal and external environments. This is consistent with previous evidences that have described numerous economic, social, psychological and behavioral challenges faced by PLWH [116–118]. For example, UNAIDS reports showed that 21% of PLWH were denied access to health care [118]. Although these problems are common among people with other chronic diseases, they are more pronounced among PLWH. There are three main reasons for this: (1) HIV is a highly stigmatized disease due to its mode of transmission, which has a strong social and cultural impact on the patient, (2) disease management requires long-term commitment from the patient, community and health system, and (3) a single problem can create multiple chains of problems [118–121]. For example, stigma and discrimination against the patient leads to poor adherence, which increases the risk of progression to AIDS, violence, and marginalization, while reducing access to education and employment [118]. Patients should be educated to be resilient and empowered to solve problems, lead productive lives and improve their quality of life. Health professionals should support and empower patients to solve problems on their own.

PLWH respond to a variety of internal and external problems with positive or negative coping mechanisms. In line with previous studies, this meta-synthesis depicted that positive coping mechanisms can include seeking support, using problem-solving strategies, living a healthy lifestyle, and developing self-compassion and mindfulness. With these skills, they can easily overcome problems and improve their quality of life [122–124]. In contrast, negative coping mechanisms, such as avoidance and escapism, denial, isolation, and substance use or addiction [117, 124], are ineffective in overcoming problems; rather, they complicate them. They usually lead to other problems including substance abuse, non-adherence to treatment, non-disclosure, anxiety, and depression, all of which affect patients' quality of life [117, 125]. It is imperative for patients to choose positive coping mechanisms when they encounter problems; this requires patients to be self-aware, self-efficacious, and have a positive outlook. Patients themselves and health care professionals should enhance skills of patients for a better quality of life.

Health care providers, according to PLWH, lack an understanding of patients' issues beyond their medical problems. They practice professional-centered care that ignores the chronicity of the disease, is inadequate to empower patients, and ignores the role of the patient, making care management unsustainable [126–128]. This type of care management has increased costs and increased patient burden on the health care system, which negatively impacts treatment outcomes [126, 128, 129)]. Recently, providers have increased their demands for patients to learn to cope with their disease and self-manage, and to learn and incorporate self-management into their service delivery [130, 131]. It is essential to follow a methodical, patient-centered approach that empowers patients having chronic disease to actively participate in daily disease management and decision making, complemented by improved provider knowledge and skills.

The desire of PLWHs is to overcome the multidimensional problems they face and live healthy lives. They have a desire to acquire relevant knowledge and skills to be the center of their own care management (self-management). Several studies show that patients play an important role in mitigating the negative effects of chronic diseases [126, 132]. Patients are in a better position to manage their chronic disease for two reasons: (1) they know their condition better than others; much of living with a chronic condition is done without external support; and (2) better treatment outcomes are achieved only when they are engaged, as they continually make decisions that affect the course of their disease. Self-management offers the opportunity to put patients at the center of their own care with full responsibility, contributing to better outcomes, higher quality of care and reduced burden on the healthcare system. Thus, the inclusion of self-management as a service component is key to increasing positive treatment outcomes for patients and reducing the burden on the healthcare system.

### Implication for practice

Our paper provides a good overview of the experiences of HIV patients and their need for self-management. We have identified possible interventions areas that will optimize the care of HIV patients, especially in developing countries. A special focus should be given during service delivery or intervention to PLWH that goes beyond the medical problems of patients. A comprehensive SM intervention package is needed to halt the negative impact of HIV and promote positive outcomes. Integration of SM intervention packages into routine service delivery and treatment guidelines, followed by capacity building of service providers, should also be considered.

Health care workers should also pay more attention to their relationship with patients, as this has a significant impact on service delivery. They should ensure that they create a safe, open environment where PLWH feel comfortable to share experiences and ask questions. In addition, empowerment workshops for PLWH should be considered. In this way, PLWH can learn about their rights and responsibilities and improve their self-management skills.

### Implication for future research

Self-management interventions for other chronic diseases, such as diabetes, hypertension, and heart failure, have been studied in LMICs and have been shown to improve physiological indicators, self-care knowledge, and quality of life in people with chronic diseases [133]. However, this practice has not been well studied among HIV patients, so the development and testing of SM interventions among PLWH is critical for the health care system in developing countries. Thus, the authors recommend exploring self-management interventions packaged with IFSMT effectiveness on improvement of the treatment outcomes among HIV patients. Furthermore, the development of tools for patient self-assessment and for health care providers to assess patients' SM practices is recommended.

### Limitations of this study

The study has some limitations that need to be mentioned. The study was initially designed as a mixed-methods review and was registered in Prospero on April 19, 2021 (ID: CRD42021247459). Later, due to the large number of qualitative studies available, we changed to a meta-synthesis of qualitative studies. A review depends on the quality and value of the included studies. While the quality of the included studies was mostly good, their individual value is mostly moderate due to small sample sizes. It must also be acknowledged that a large proportion of the included studies (70%) were conducted in Africa and the applicability to other developing countries outside the African continent may be limited.

## Conclusion

PLWH have a desire to live healthy, productive, and quality lives; however, they face multiple problems that come from both themselves and the external environment. In addition, because of their lack of knowledge and self-efficacy skills, PLWH face more complicated problems as a result of their responses to the primary problems. These complicated and multifaceted problems, coupled with a lack of support and proper chronic care management by the health facility, limit functionality, reduce productivity, worsen health status, and affect the quality of life of the patient. In addition, the negative impact extends to the family or society, resulting in increased burden and lost productivity, and to the health care system, resulting in increased burden.

In order to achieve better and more sustainable disease management and to improve outcomes and quality of life, patients should be engaged and empowered to manage their own care. Patients are in a better position than healthcare providers to monitor their chronic disease on a daily basis, and better outcomes can be achieved if they are engaged. Self-management practices offer us a better opportunity to put the patient at the center of his or her own care, with appropriate guidance from well-informed providers. In addition, both patients and providers have called for a more structured and comprehensive package of self-management interventions to overcome barriers and ensure a fulfilling life. The development of tools for patient and provider self-assessment of patients' SM practices, and the development and testing of SM interventions among PLWH, are critical to the health care system in developing countries.

### Supplementary Information

**Additional file 1.** Self-efficacy skills andabilities.

## Data Availability

Not applicable.
