# Supplementary material for: Experiences of people living with HIV in low- and middle-income countries and their perspectives in self-management: a meta-synthesis
Source: AIDS Res Ther. 2024 Jan 31;21:7. doi: 10.1186/s12981-024-00595-7 (PMC10829476; doi:10.1186/s12981-024-00595-7)
Supplement: Supplementary file 1 — Additional file 1. Self-efficacy skills andabilities. [file 12981_2024_595_MOESM1_ESM.docx]

**Search strategies**

**Initial search in March 2020**

Embase, Pubmed and the Cochrane database.:

“people living with HIV/ HIV patients/ PLWH/ HIV” and “self-management/ selfmanagement/ self-care/ self-evaluation/ self-monitoring” and “interviews/ interview/ questionnaire/ observational study/ focus group/ focus groups”.

**Second search between 2020 to 2022**

PubMed

((((((((HIV) OR (HIV patients)) OR (PLWH)) OR (HIV) ) AND (self-management)) OR (selfmanagement)) OR (self-care)) OR (self-evaluation)) OR (self-monitoring) AND (2020:2023[pdat]) AND ((humans[Filter]) AND (adolescent[Filter] OR alladult[Filter] OR youngadult[Filter] OR adult[Filter] OR middleagedaged[Filter] OR middleaged[Filter] OR aged[Filter] OR 80andover[Filter]) AND (2020:2022[pdat]))

EBSCOS: CINHAL, MEDLINE, Psychinfo, health and psychology

AB self-management OR AB selfmanagement OR TI self-care OR TI self-evaluation OR TI self-monitoring AND TI HIV OR TI PLWH OR TI human immunodeficiency virus

**Limiters** - Published Date: 20200101-20221231

**Narrow by SubjectMajor:**- patient education, - hiv seropositivity,- mobile applications, - health promotion, - self-efficacy, - primary health care, - patient compliance, - substance use disorders, - attitude to health, - viral load, - health knowledge, - human immunodeficiency virus, - preventive health care, - support, psychosocial, - health services accessibility, - health behavior, - treatment outcomes, - medication compliance, - stigma, - quality of life, - women's health, - risk assessment. - patient attitudes, - self-management, - self care, - hiv-positive persons, - hiv infections,- English, - aged, 80 & over, - aged: 65+ years, - adolescent: 13-18 years, - middle aged: 45-64 years, - all adult, - adult: 19-44 years,

**Search modes** - Boolean/Phrase

**Google scholar:**

self-management OR selfmanagement OR self-care OR self-evaluation OR self-monitoring AND HIV OR PLWH OR human immunodeficiency virus
